# *Malus* Hosts–*Erwinia amylovora* Interactions: Strain Pathogenicity and Resistance Mechanisms

**DOI:** 10.3389/fpls.2019.00551

**Published:** 2019-04-26

**Authors:** Ofere Francis Emeriewen, Thomas Wöhner, Henryk Flachowsky, Andreas Peil

**Affiliations:** Federal Research Centre for Cultivated Plants, Institute for Breeding Research on Fruit Crops, Julius Kühn-Institut (JKI), Dresden, Germany

**Keywords:** apple, AvrRpt2_EA_, effectors, SNP, susceptible, virulence, gene-for-gene, wild apple

## Abstract

The bacterium, *Erwinia amylovora*, deposits effector proteins such as AvrRpt2_EA_ into hosts through the type III secretion pathogenicity island to cause fire blight in susceptible *Malus* genotypes. A single nucleotide polymorphism in the AvrRpt2_EA_ effector plays a key role in pathogen virulence on *Malus* hosts by exchanging one cysteine to serine in the effector protein sequence. Fire blight resistance quantitative trait loci (QTLs) were detected in a few apple cultivars and wild *Malus* genotypes with the resistance of wild apples generally found to be stronger than their domestic relatives. The only candidate and functionally analyzed fire blight resistance genes proposed are from wild apple genotypes. Nevertheless, the aforementioned AvrRpt2_EA_ SNP and a couple of effector mutants of *E*. *amylovora* are responsible for the breakdown of resistance from a few *Malus* donors including detected QTLs and underlying *R*-genes. This review summarizes a key finding related to the molecular basis underpinning an aspect of virulence of *E*. *amylovora* on *Malus* genotypes, as well as mechanisms of host recognition and specificity, and their implications on the results of genetic mapping and phenotypic studies within the last 5–6 years. Although the knowledge gained has improved our understanding of the *Malus*–*E. amylovora* system, more research is required to fully grasp the resistance mechanisms in this genus especially as they pertain to direct interactions with pathogen effectors.

## Introduction

### Economic Importance of Fire Blight and Host Immunity

*Erwinia amylovora* (Burrill) (Winslow et al., 1920) incites fire blight disease in the genus *Malus* as it does other genera belonging to the Rosaceae family. The bacterium invades hosts primarily through flowers or wounds on vegetative tissues and migrates internally to other infection sites causing blossom, shoot, and rootstock blights (Peil et al., 2009). This erratic disease is the most feared bacterial disease especially for apples and pears, the two most economically important members of Rosaceae. Commercial cultivars of the domesticated apple (*Malus domestica* Borkh.) are highly susceptible, although some show low susceptibility (Sobiczewski et al., 2011). Resistance sources are found mostly in wild species having unmarketable fruit quality (Kellerhals et al., 2017). The economic damages following fire blight outbreaks are estimated in millions of Dollars wherever they have been reported (Hasler et al., 2002; Norelli et al., 2003). These huge costs stem from the destruction of trees due to the ability of the pathogen to completely destroy entire orchards of pome fruits in a single growing season, as well as antibiotic application as a control measure especially in the United States. In Europe, economic damages from fire blight comprise mostly of the production costs, since antibiotic application as a control measure is either strictly regulated or completely forbidden.

Therefore, fire blight resistance breeding strategy that focuses on the basic molecular mechanisms of host resistance is increasingly important due to the inadequacies of other control strategies.

*Erwinia amylovora* (*Ea*) can adopt a biotrophic or necrotrophic lifestyle, since it invades living *Malus* hosts yet could overwinter in dead apple leaves (Sobiczewski et al., 2014, 2017). Like other plant-pathogen models (See: Dodd and Rathjen, 2010), *Ea* manipulates host defense mechanisms to cause disease, while *Malus* host immunity/resistance depends on their capability to recognize *Ea* elicitors (e.g., effector proteins) through different molecular strategies. In general, surface receptor proteins known as pattern recognition receptors (PRRs) recognize conserved microbial patterns (pathogen-associated molecular patterns; PAMP), e.g., bacterial flagellin, lipopolysaccharide, in PTI—pattern triggered immunity (Couto and Zipfel, 2016). On the other hand, intracellular receptors could recognize pathogen effector proteins during invasion of host cells in effector triggered immunity (ETI; Toruño et al., 2016). In principle, ETI and PTI elicit similar responses in hosts; however, only the former involves hypersensitive response (HR)—a form of localized cell death. HR is controlled by hypersensitive response and pathogenicity genes (*hrp*) (Khan et al., 2012) of the so-called pathogenicity island (PAI) (Oh and Beer, 2005).

Knowledge of *Malus*–*Ea* interaction is crucial for breeding durable resistant apples, and past and current works have been done with regard to characterizing pathogenicity genes of *Ea* (reviewed in Oh and Beer, 2005; Khan et al., 2012; Malnoy et al., 2012), as well as breeding for resistance (Peil et al., 2009). This review focuses on works that have contributed to our understanding as it pertains to *Malus*–*Ea* interaction and host resistance mechanisms since 2013 till date.

## *Malus* Host Resistance: Strains vs. Resistance

Vogt et al. (2013) and other studies (Table 1) provided valuable insights into *E*. *amylovora* pathogenicity and *Malus* resistance. The *avrRpt2_EA_* effector gene of several *Ea* strains was sequenced and detected a single nucleotide polymorphism (SNP), which translated into a single substitution of amino acid cysteine (C-allele) with serine (S-allele) at position 156 of the protein sequence. Meanwhile, resistance to fire blight was thought to be quantitatively controlled in *Malus*, and prior to 2013, two fire blight resistance quantitative trait loci (QTLs) with minor effects were described in apple cultivars—“Fiesta” (Calenge et al., 2005) and “Florina” (Le Roux et al., 2010) and three QTLs with major effects in wild apple genotypes *Malus* ×*robusta* 5 (Mr5) (Peil et al., 2007; Gardiner et al., 2012), *M. floribunda* 821 (Mf821), and the ornamental cultivar “Evereste” (Durel et al., 2009). However, in Mr5, a single resistance gene was shown to underlie the QTL region. For these studies, *Ea* strain CFBP1430 from the French collection was used for artificial shoot inoculations; the exception is in *Malus* ×*robusta* 5 (Mr5), where Ea222 was used. Vogt et al. (2013) showed that both Ea222 and CFBP1430 encode for the C-allele of the *avrRpt2_EA_* effector of *E*. *amylovora*. Interestingly, strains bearing the C-allele are avirulent to Mr5, whereas strains bearing the S-allele are not, thus supporting the strain specificity of Mr5 fire blight resistance (Peil et al., 2011).

**Table 1:** Examples of fire blight studies in *Malus* and *Erwinia amylovora* strain types used.

Studies	Pathogen strains	Type	*Malus* genotypes/cultivars
Peil et al., 2007	Ea222	C-allele	*Malus* ×*robusta*5
Peil et al., 2008	Ea3049	S-allele	
Wöhner et al., 2014	ZYRKD3–1^*^	Mutant	
Emeriewen et al., 2014a,b	Ea222	C-allele	*Malus fusca* MAL0045
Emeriewen et al., 2015	Ea3049	S-allele	
Emeriewen et al., 2017b	ZYRKD3-1	Mutant	
Wöhner et al., 2017	LA635, LA637	S-alleles	
Emeriewen et al., 2017a	Ea222	C-allele	*Malus* ×*arnoldiana* MAL0004
	Ea3049	S-allele	
Wöhner et al., 2017	Ea1189	C-allele	
	Eop1^+^	Mutant	
Durel et al., 2009	CFBP1430	C-allele	“Evereste”
Wöhner et al., 2017	Ea1189	C-allele	
	Ea3049	S-allele	
	LA635	S-allele	
	LA637	S-allele	
	Eop1	Mutant	
Durel et al., 2009	CFBP1430	C-allele	*Malus floribunda* 821
Wöhner et al., 2017	Ea1189	C-allele	
	Ea3049	S-allele	
	LA635	S-allele	
	LA637	S-allele	
	Eop1	Mutant	
Harshman et al., 2017	Ea153n	-	*Malus sieversii*
Desnoues et al., 2018	Ea273	C-allele	
Calenge et al., 2005	CFBP1430	C-allele	“Fiesta”
Le Roux et al., 2010	CFBP1430	C-allele	“Florina”
Desnoues et al., 2018	Ea273	C-allele	“Royal Gala” (Tenroy)
Wöhner et al., 2017	Ea1189	C-allele	
	Ea3049	S-allele	
	LA635	S-allele	
	LA637	S-allele	
	Eop1	Mutant	

C-and S-alleles: Single nucleotide polymorphism at position 156 of the avrRpt2_EA_ effector leading to a switch from Cysteine (C-allele) to Serine (S-allele) amino acids (Vogt et al., 2013).

* Strain with disruption in the *avrRpt2_EA_* gene (Vogt et al., 2013).

+ Ea1189 Eop1 deletion mutant.

Vogt and colleagues proved that strains encoding for the S-allele were more aggressive for different apple cultivars, and Mr5 itself - a major source of fire blight resistance, but not on other wild apple accessions of *Malus fusca* and *Malus baccata*. Consequently, the suspicion that the fire blight resistance QTL of Mr5 on linkage group 3 (LG3) is broken down by the highly aggressive S-allele encoding Canadian strain Ea3049 (Peil et al., 2011), was confirmed by Wöhner et al. (2014). In addition, minor QTLs contributing to fire blight resistance in Mr5 were detected (Peil et al., 2011; Wöhner et al., 2014), with one in particular on LG7 found to improve resistance of LG3 QTL (Wöhner et al., 2014). *Erwinia amylovora* strain specificity in *Malus* begs the question “**Can reduced susceptibility of apple cultivars be considered as resistance**?” It could be argued that the fire blight QTLs of apple cultivars like “Fiesta” and “Florina” actually control reduced susceptibility to the disease rather than resistance, given that fire blight QTLs in those apple cultivars have been shown to only explain minor effects to *Ea* strains considered as less aggressive.

A few novel fire blight minor QTLs were identified in a “Royal Gala” × *M. sieversii* population (Desnoues et al., 2018). However, two QTLs appear to be the same as QTLs of “Florina” (Le Roux et al., 2010) and “Co-op16” × “Co-op17” (Khan et al., 2013) on chromosomes 10 and 15, respectively, which were identified with strains Ea273 and CFBP140, which encode the C-allele of AvrRpt2_EA_ effector protein (Vogt et al., 2013). While strong fire blight resistance of some accessions of *M. sieversii* was reported (Harshman et al., 2017), it is unsurprising that the resistance QTLs/levels of *M. sieversii* accession PI 613981 (Desnoues et al., 2018) are similar to that of apple cultivars “Florina” and “Nova Easygro” (Le Roux et al., 2010), since *M. sieversii* is the primary progenitor of *M. domestica*. Similarly, the alleles of QTLs in coupling with resistance inherited by the apple cultivar “Enterprise” and a breeding clone X-6398 (van de Weg et al., 2018) were found to be identical to “Fiesta” (Calenge et al., 2005), with both studies finding epistatic effects, suggesting similar mechanism of reduced fire blight susceptibility. Nonetheless, reduced susceptibility mechanism (small effects) of cultivars to *E*. *amylovora* is a defense mechanism capable of contributing to the overall goal of durable resistance by slowing down the effect of mutations of the pathogen that may overcome resistance. However, little is known about the reactions of QTL donor cultivars to strains with stronger pathogenicity (Table 1). Moreover, testing populations used to detect QTLs in cultivars (not just donor parents) with AvrRpt2_EA_-S-allele-encoding strains will confirm the strength of such QTLs and importantly determine if transgressive individuals (i.e., progenies more resistant or significantly less susceptible than the QTL donor parent) could be obtained. Transgressive progenies would be more worthwhile to use in breeding programs.

In the *Malus*–*Ea* system, non-strain-specific (i.e. broad spectrum) resistance appears to be the exception rather than the rule. The fire blight resistance of the *M. fusca* genotype MAL0045 (hereafter referred to as *M. fusca*, Emeriewen et al., 2014a) appears to be broad spectrum. This genotype is the source of *Mfu10*—a resistance QTL mapped on LG10 that was detected with strain Ea222 (Emeriewen et al., 2014b). C- and S-allele strains of the pathogen tested on *M. fusca* were unable to overcome neither its resistance nor *Mfu10* (Vogt et al., 2013; Emeriewen et al., 2015). Although the S-allele strain Ea3049 could not overcome *Mfu10*, the population was adversely affected (Emeriewen et al., 2015), suggesting the possibility of a second putative genetic factor contributing to the resistance of *M. fusca*. A situation where *M. fusca* is highly resistant to such an aggressive S-type strain but almost half of its progenies were found to be highly susceptible indicates that such putative factor could be homozygous recessive in this crabapple accession. However, a “less” resistant reaction was elicited by *M. fusca*, following fire blight disease assessment with two other S-allele strains LA635 and LA637 (Wöhner et al., 2017). Both strains were shown to not only possess the single nucleotide polymorphism in the type III effector AvrRpt2_EA_ at position 156 but also an *rpsL* chromosomal mutation responsible for a high degree of streptomycin resistance (Smits et al., 2014). It is unclear whether the combination of the unique mechanisms arising from genomic mutations controlling drug resistance and pathogenicity contributes to the aggressive nature of LA635 and LA637 strains. Clearly, the SNP in AvrRpt2_EA_ is not the only contributory virulence factor in S-allele strains.

**Figure 1:**
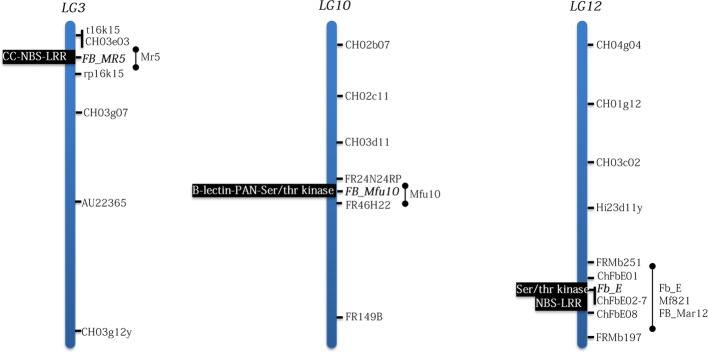
Positions of fire blight (candidate) genes and their domains identified in *Malus* following positional cloning studies. Bar represents positions of mapped QTLs on the linkage groups. Although no candidate genes have being proposed for *M*. ×*arnoldiana* and *M. floribunda* 821, their respective QTLs, *FB_Mar12* and *Mf12* map in the region where *Fb_E* is located on LG12. LG=linkage group.

Another source of strong fire blight resistance is the wild apple genotype *Malus* ×*arnoldiana* MAL0004 initially mentioned as *M. baccata* (Vogt et al., 2013; Peil et al., 2014). This wild apple is the source of a strong and stable fire blight resistance QTL (*FB_Mar12*) mapped on LG12 (Emeriewen et al., 2017a). *FB_Mar12* detected simultaneously with both the C-allele (Ea222) and S-allele (Ea3049) strains of *E*. *amylovora* was mapped at a similar position on chromosome 12 like the QTLs of Mf821 and “Evereste” (Durel et al., 2009), suggesting that all three QTLs could be allelic (Figure 1). Although no additional evidence of their allelic nature exists, however, Wöhner et al. (2017) provided strong evidence that the resistance mechanisms of all three wild apples are in fact different. While all three donors of each QTL, i.e., MAL0004, Mf821, “Evereste,” elicit strong resistance reaction to a C-allele strain Ea1189, such resistance reaction in “Evereste” and Mf821 appears to be overcome by Ea3049, LA635, and LA637, bearing the S-allele but not in the *M*. ×*arnoldiana* accession (Wöhner et al., 2017). Thus, *FB_Mar12* and *Mfu10* are the most stable and durable fire blight resistance QTLs in *Malus* so far.

## *Malus*–*Erwinia Amylovora* Interactions: The Role of Effector Mutants

Since the *Malus*–*Ea* pathosystem is characterized by the ability of the host to activate defense responses upon recognition of effectors secreted by *Ea*, the use of effector protein knock-out strains is an important tool for deciphering *Malus*–*Ea* interactions. The pathogen possesses the *hrp*-T3SS (*hrp* type III secretion) system, releasing type III effectors essential for pathogenicity, e.g., DspE, AvrRpt2_EA_, Eop1, Eop3, and the exopolysaccharide amylovoran (*ams*), into the host cell. However, T3SS chaperones play critical roles in translocating effector proteins (Castiblanco et al., 2018); other genes (overviewed in McNally et al., 2015) could determine virulence in *Ea*. Gene-for-gene interactions were shown in Mr5 (Vogt et al., 2013) and partially in Mf821 and “Evereste” (Wöhner et al., 2017). An *avrRpt2_EA_* mutant strain ZYRKD3-1 broke down the resistance of Mr5, thus indicating the first gene-for-gene interaction in *Malus* and the avirulence role of *avrRpt2_EA_* in Mr5–*Ea* pathosystem (Vogt et al., 2013). AvrRpt2_EA_ was further shown to play no such roles in the examined *M. fusca*, *M*. ×a*rnoldiana* and Mf821 systems (Vogt et al., 2013; Wöhner et al., 2017; Emeriewen et al., 2017b). Further, both the C- and S-alleles of AvrRpt2_EA_ effector expressed in *Nicotiana benthamiana* induced cell death as did the *Pseudomonas syringae* effector AvrRpt2 (Vogt et al., 2013), thus suggesting an interaction between Mr5 and *Ea* that mimics the RPS2–*Arabidopsis thaliana* model (Axtell and Staskawicz, 2003). In addition to being an avirulence factor in Mr5, the role of *avrRpt2_EA_* as a virulence factor in susceptible hosts was demonstrated by Schröpfer et al. (2018): transgenic apple lines expressing *avrRpt2_EA_* developed typical fire blight symptoms. Also, these apple lines resulted in increased expressions of *PR-1* gene, thus inducing salicylic acid–dependent defense response (Schröpfer et al., 2018). Deletion of *eop1* effector of *E*. *amylovora* induced fire blight in Mf821 and “Evereste” but not in *M*. ×a*rnoldiana* (Wöhner et al., 2017), thus confirming that the resistance mechanism of *M*. ×a*rnoldiana* is different although their QTLs are closely located on LG12 (Figure 1). Furthermore, that *eop1* breaks down the resistance of Mf821 and “Evereste” is strong evidence of another gene-for-gene interaction in *Malus* (Wöhner et al., 2017). Comparative mapping studies with Eop1 mutant and wild type strains on Mf821 and “Evereste” will confirm the gene-for-gene interactions or otherwise.

## *Malus* Fire Blight Resistance (Candidate) Genes

Identifying fire blight genes and the subsequent functional analyses are crucial in understanding resistance mechanisms in *Malus*. Only map-based cloning approach has resulted in credible fire blight candidate genes till date (Figure 1). The first set of fire blight resistance candidate genes in *Malus* (Parravicini et al., 2011) was hypothesized to function in a similar mode as the well-characterized *Pto*-mediated resistance to bacterial speck in tomato (Pedley and Martin, 2003). Although, the candidate genes from the fire blight QTL region of the ornamental apple cultivar, “Evereste,” showed homology to the *Pto/Prf* system (Parravicini et al., 2011), it is yet to be determined if these genes actually confer resistance to fire blight in complementing studies.

The guard hypothesis was postulated for *FB_MR5* identified in the Mr5 fire blight QTL region (Fahrentrapp et al., 2013). Although multiple genes were suggested in this region to contribute to resistance (Gardiner et al., 2012), *FB_MR5* was proposed as the only candidate and is a single *R*-gene belonging to the class of plant disease *R*-genes families encoding for nucleotide-binding site (NBS), a C-terminal leucine rich repeat (LRR), and a coiled coil domain (CC). Like RPS2 being triggered on recognizing the disturbance of *Arabidopsis thaliana* RIN4 protein by AvrRpt2 type III effector avirulence protein from *Pseudomonas syringae* (Mackey et al., 2003), AvrRpt2_EA_, an analog in *E*. *amylovora* (Zhao et al., 2006), was hypothesized to target the homolog of RIN4 in Mr5 (Fahrentrapp et al., 2013). However, protein-protein interaction analyses *via* yeast two-hybrid assays failed to detect any interaction between RIN4 from Mr5 and either of the fire blight resistance protein FB_MR5 from Mr5 or the effector protein AvrRpt2_EA_ from *E*. *amylovora* (Vogt, 2018). A recent study analyzing the protein structure of AvrRpt2_EA_, however, suggests a direct interaction with FB_MR5 (Bartho et al., 2019). Importantly, *FB_MR5* confers resistance to transgenic lines of an otherwise susceptible cultivar “Gala,” expressing this gene that was inoculated with Ea222 (Broggini et al., 2014)_._ Transgenic lines expressing *FB_MR5* inoculated with the mutant strain with disruption of the corresponding avirulence gene (*avrRpt2_EA_*) also demonstrated the gene-or-gene relationship found in Mr5 (Broggini et al., 2014).

In *M. fusca*, a candidate gene (*FB_Mfu10*) encoding Bulb-lectin, PAN/apple and serine/threonine kinase domains, was shown to underlie *Mfu10* (Emeriewen et al., 2018). The resistance model in *M. fusca* is so far not obvious. If indeed *FB_Mfu10* confers broad spectrum resistance, the mechanism could be similar to the proposed zigzag model of PRRs (pathogen recognition receptors) as first line of active defense (Jones and Dangl, 2006). As reviewed elsewhere (Zipfel, 2014), PRRs are either receptor kinases (RKs) or receptor-like proteins (RLPs). Is *FB_Mfu10* a potential PRR? What is the corresponding pathogen recognition molecular pattern—*ams* or effectors? Effector proteins may act as PAMPs (Thomma et al., 2011), and some may target PTI rather than ETI (Jones and Dangl, 2006). Nevertheless, the lack of the AvrRpt2_EA_ effector increasing average disease in *M. fusca* progeny (Emeriewen et al., 2017b) at least indicates AvrRpt2_EA_ effector triggered response.

Further, the roles of lectin and PAN/apple domains in mediating protein-carbohydrate and protein–protein interactions and defense signaling have been shown (Lannoo and Van Damme, 2014; Kim et al., 2015). To date, no *Ea* effector mutant able to break the resistance of *M. fusca* is reported to suggest a gene-for-gene interaction. Functional tests of *FB_Mfu10* in transgenic lines will therefore improve our understanding of the fire blight resistance of this crabapple.

## Concluding Remarks

*Malus*–*Ea* interaction may be governed by a distinct complementary interaction between pathogen avirulence (*Avr*) effectors and alleles of the corresponding host resistance (*R*) locus or gene, but other factors and models cannot be excluded. The identification of a SNP in the AvrRpt2_EA_ effector responsible for strain pathogenicity in Mr5, but also virulence in other *Malus* hosts (Vogt et al., 2013), was a major breakthrough in understanding this system. Molecular breeding for durable and broad spectrum resistance to fire blight should take into cognizance the virulence factors of the pathogen and the reaction of *Malus* hosts. The use of S-allele strains of *E. amylovora* should be the benchmark in identifying strong and durable resistance gene sources. It is also imperative to investigate resistance donors from apple cultivars using S-allele strains. However, further studies on host-pathogen interaction are still required to achieve durable resistance. Knockout of genes of the pathogenicity island of different *E*. *amylovora* strains and other *E*a mutations, which have led to the discovery of more virulence factors (Ramos et al., 2013, 2015; Klee et al., 2018), will improve our understanding of the pathogenic process.

Presently, only one functionally analyzed fire blight resistance gene in *Malus* from Mr5 (Broggini et al., 2014) is known, although the resistance is overcome. The others are yet to be proven candidate genes from “Evereste” (Parravicini et al., 2011) and *M. fusca* (Emeriewen et al., 2018). Since the entire resistance gene sources are from wild apples with undesirable fruit qualities, coupled with the ability of the pathogen to overcome resistance over time, the potentials of susceptibility (*S*) genes have been suggested (Reviewed in van Schie and Takken, 2014). Taking into account, the HrpN-interacting protein of *Malus* (HIPM) was investigated as a potential fire blight *S* gene (Campa et al., 2018). These authors showed that *HIPM*-silenced lines of the apple cultivar Galaxy® showed reduced susceptibility to strain Ea273, a C-allele strain. Similarly, with a C-allele strain, CFBP1430, susceptible response of “Golden Delicious” cultivar, was studied *via* RNA-seq, leading to the identification of differentially expressed genes (Kamber et al., 2016). It remains unclear what role *S*-genes will play in the long term especially considering the evolutionary potential of the pathogen. What is clear, however, is the need to functionally analyze other resistance candidate genes, identify more resistance gene sources, and perform more resistance protein and effector protein interaction studies.

## Author Contributions

All the authors conceptualized this review and discussed the approach. OFE wrote the manuscript and all authors edited and finally approved the manuscript.

### Conflict of Interest Statement

The authors declare that the research was conducted in the absence of any commercial or financial relationships that could be construed as a potential conflict of interest.
